# New Insights in Glucocorticoid Receptor Signaling—More Than Just a Ligand-Binding Receptor

**DOI:** 10.3389/fendo.2017.00016

**Published:** 2017-02-06

**Authors:** Karin Scheschowitsch, Jacqueline Alves Leite, Jamil Assreuy

**Affiliations:** ^1^Department of Pharmacology, Universidade Federal de Santa Catarina, Florianópolis, Brazil; ^2^Department of Pharmacology, Institute of Biomedical Sciences, Universidade de São Paulo, São Paulo, Brazil

**Keywords:** glucocorticoid receptor, glucocorticoids, selective glucocorticoid receptor modulators, signaling pathways, nuclear translocation

## Abstract

The clinical use of classical glucocorticoids (GC) is narrowed by the many side effects it causes and the resistance to GC observed in some diseases. Since the great majority of GC effects depend on the activation of a glucocorticoid receptor (GR), many research groups had focused to better understand the signaling pathways involving those receptors. Transgenic animal models and genetic modifications of the receptor brought a huge insight into GR mechanisms of action. This in turn opened a new window for the search of selective GR modulators that ideally may have agonistic and antagonistic combined effects and activate one specific signaling pathway, inducing mostly transrepression or transactivation mechanisms. Another important research field concerns to posttranslational modifications that affect the GR and consequently also affect its signaling and function. In this mini review, we discuss many of those aspects of GR signaling, as well as findings like the ligand-independent activation of GR, which add another layer of complexity in GR signaling pathways. Although several recent data have been added to the GR field, much work has yet to be done, especially to find out the biological relevance of those alternative GR signaling pathways. Improving the knowledge about alternative GR signaling pathways and understanding how these pathways intercommunicate and in which situations they are relevant might help to develop new strategies to take benefit of it and to improve GC or other compounds efficacy causing minimal side effects.

## Introduction

### Importance of Glucocorticoids (GC) in Controlling Inflammation

Glucocorticoids (cortisol in humans and corticosterone in rodents) are steroid hormones [revised in Ref. (1)] involved in several physiological functions and in controlling inflammation (2–5). From their discovery (6, 7) until the present day, GC are considered the most effective anti-inflammatory drugs and one of the most widely prescribed drug classes worldwide (8–12).

Despite their potent anti-inflammatory effects, steroids cause relevant side effects when used for longer periods and at high doses (13–16), limiting their use and reducing adherence to treatment. Therefore, understanding the signaling mechanisms and pathways related to GC and their receptor [glucocorticoid receptor (GR)] is essential to provide the basis for the development of new selective glucocorticoid receptor modulators (SEGRMs) (17, 18). SEGRMs are expected to present the same or better efficacy compared to classical steroids, but causing minimal side effects (8, 14, 19–21). The present mini review will discuss advances in GR signaling pathways looking for a better comprehension about the beneficial and harmful effects of endogenous and exogenous GC, especially in inflammation.

## The GR

**Figure 1:**
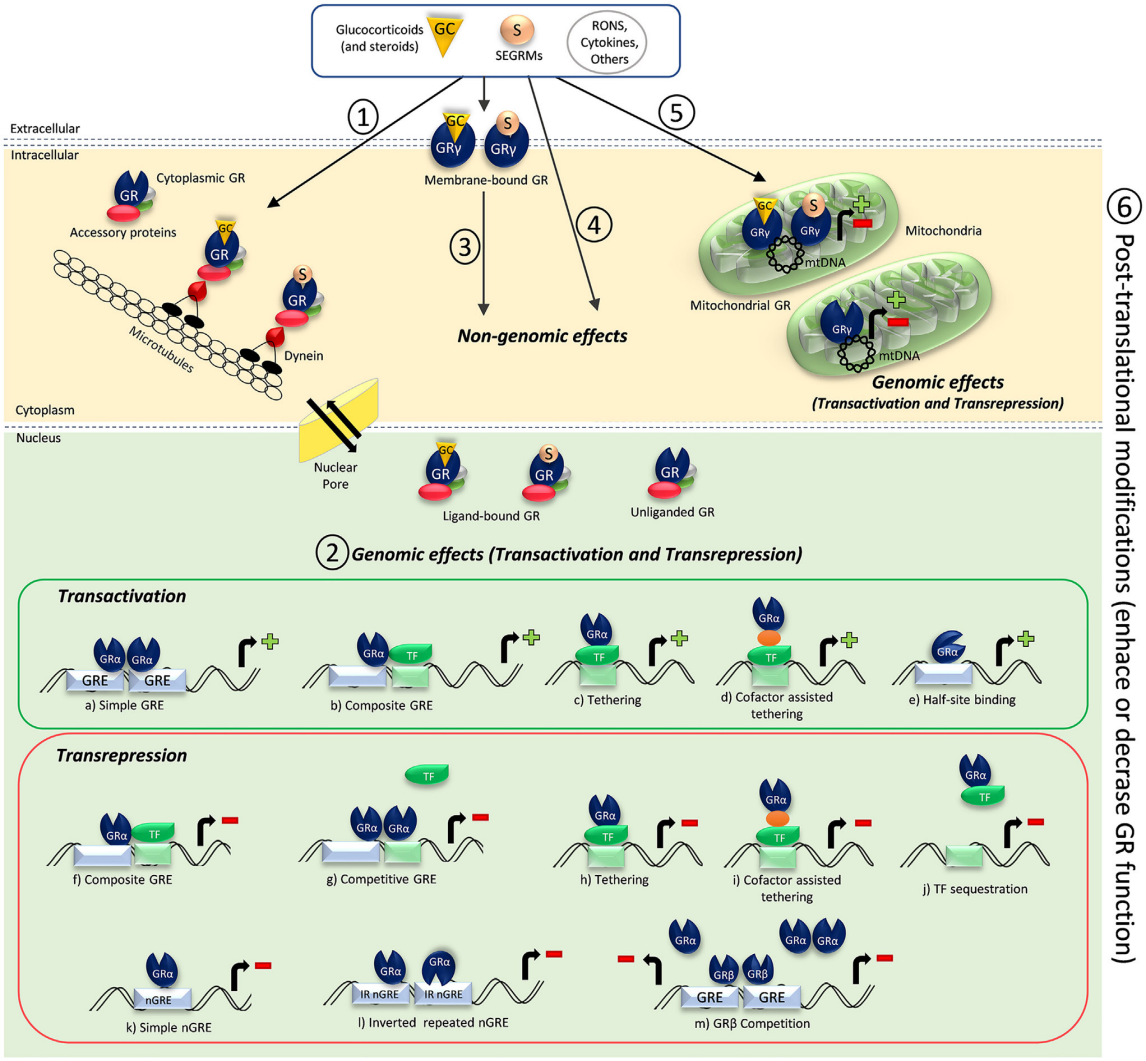
**Schematic illustration of glucocorticoid receptor (GR) activation and GR-mediated mechanisms of action**. (1) Cytoplasmic GR resides in the cytoplasm complexed with accessory proteins and present high affinity to ligands. Once ligands like glucocorticoids and other steroids or selective glucocorticoid receptor modulators (SEGRMs) bind to the cytoplasmic GR, the GR complex interacts with dynein and is transported along microtubules to a nuclear pore. Interaction with importins and nucleoporins of the nuclear pore allow the GR complex to enter the nucleus, dissociate from chaperones, and induce genomic effects. Dissociated chaperones and GR constantly shuttle between the nucleus and the cytoplasm through the nuclear pore. Reactive oxygen and nitrogen species (RONS), some cytokines, other substances, and conditions like shear stress can induce unliganded GR nuclear translocation, which seem to be cytoskeleton independent. However, unliganded GR nuclear translocation is still not completely understood. (2) Ligand-bound GR, and sometimes unliganded GR, can induce genomic effects through direct or indirect transactivation or transrepression mechanisms. GRα homodimers binding to glucocorticoid-responsive elements (GRE) **(A)**, monomeric GRα DNA binding in a concerted manner with another transcription factor (TF) **(B)**, direct **(C)** or indirect **(D)** binding of GRα onto a TF, and recently demonstrated monomeric GRα half-site binding **(E)** can result in promoter activation and gene expression. GR-negative regulation of gene transcription can occur by monomeric GRα DNA-binding crosstalk with another TF **(F)**, GRα homodimers competition for an overlapping binding site **(G)**, direct **(H)** or indirect **(I)** binding of GRα onto a TF, sequestration of a DNA-bound TF **(J)**, direct binding of monomeric GRα onto a negative GRE (nGRE) **(K)**, two monomeric GRα binding with inverted polarities to inverted repeated nGREs **(L)**, or GRβ competition for an overlapping GRE, impairing GRα binding **(M)**. (3) Ligands and other substances also can bind and interact with membrane-bound GR (claimed to be a GRγ isoform), causing fast non-genomic effects. (4) Ligands, particularly steroids in high concentrations, can induce non-genomic effects through GR-independent mechanisms of action. (5) Ligands and other substances can bind to mitochondrial GR, which is also suggested to be a GRγ isoform. Ligand-bound and unliganded mitochondrial GR induce genomic effects when bound to the mitochondrial DNA (mtDNA), and those effects are important to regulate mitochondrial functions and energy metabolism. (6) Posttranslational modifications can affect GR activation and function in all stages, enhancing or decreasing its function.

The main actions of GC occur through the activation of GRs (NR3C1), which are transcription factors (TFs) belonging to the superfamily of nuclear receptors and are usually activated by ligands (6, 14, 22). GR is composed of three major functional domains, namely the *N*-terminal transactivation domain (NTD), the central DNA-binding domain (DBD), and the *C*-terminal ligand-binding domain (LBD) (23, 24). In the absence of ligands, GR is predominantly found in the cytoplasm complexed with accessory proteins (hsp90, hsp70, hsp56, p23, and immunophilins) and is kept in a conformation of high-binding affinity to GC (25, 26) (see Figure 1: 1). GR can actively shuttle between cytoplasm and nucleus, being the balance rate of nuclear import and export which determines the receptor cellular location (27–29). Increases in receptor density affect its conformation and location and may cause ligand-free dimerization that facilitates the subsequent binding of ligands, thus bypassing dimerization-dependent mechanisms of action (30).

One of the first proposed signaling pathways for GR was that the binding of a ligand to the LBD of monomeric GR would induce its nuclear localization sequence (NLS) exposure. Then, accessory proteins would dissociate from the monomeric receptor, allowing it to dimerize and translocate along microtubules to the nucleus. There, GR dimers would bind to DNA through their DBD to exert their effects (27, 31–33).

Further evidence showed that ligand binding triggers NLS exposure due to the replacement of immunophilin FKBP51 by FKBP52, which interacts with dynein, carrying the whole monomeric GR complex along microtubules to a nuclear pore. FKBP52 also facilitates the entry of GR monomeric complex into the nucleus, as well as exporting the accessory proteins back to the cytoplasm *via* importins (25, 34–37) (see Figure 1: 1). Once in the nucleus, monomeric GR can assume different conformations depending on the glucocorticoid-responsive elements (GREs). GR monomer can recruit another monomer to form a GR homodimer on DNA, through distinct hydrophobic motifs of the LBD (38). Nevertheless, the subcellular compartment for GR dimerization is still in debate, and the GR binding regions used by specific GREs are still unclear (39). Therefore, more studies aiming to create predictive models for GR activity may help the development of new compounds.

Regarding GR nuclear translocation, it is suggested that different ligands can induce the exposure of one of the two so far described NLS for GR, influencing its nuclear translocation speed (27, 28, 34, 40). Classical GR ligands are suggested to induce NLS-1 exposure, which interacts with importins and nucleoporins, leading to rapid nuclear translocation (within 4–6 min). Shuttling of unliganded GR depends on NLS-1 interaction with importin-alpha. On the other hand, NLS-2 exposure is strictly ligand dependent and mediates slower GR nuclear translocation (45 min–1 h) (28, 36, 41).

## Transgenic Animal Models

Since GR was cloned (42), the development of animals and cells with full or partial GR depletion and with different GR mutations allowed the study of the role of GR. The first GR knockout mice (GR^null^) presented severe lung developmental abnormalities and died shortly after birth (43). On the other hand, animals that overexpress GR are resistant to septic shock (44). Organ-specific GR depletion have shown that (a) hepatic GR is responsible for gluconeogenesis and production of postnatal insulin-like growth factor-1 (45, 46); (b) GR depletion in the central nervous system leads to numerous behavioral abnormalities (47); (c) mice with specific depletion of GR in macrophages, neutrophils (48–50), or endothelial cells (51) are more sensitive to pro-inflammatory stimuli, evidencing the important role for GR in these cells for the physiological control of inflammation.

Development of a mutation in the second zinc finger of DBD (52) (called GR^dim^) prevented GR homodimerization on most tandem GREs (53). Notwithstanding that this mutation strongly attenuates GR dimerization and impairs GR transcription activity from tandem GREs (21, 54, 55), it does not completely abrogate transactivation mechanisms, since it was recently discovered that it depends on promoter contexts (56). Furthermore, DNA motifs specify the genomic occupancy of monomeric GR and interfere with the availability of GR dimers binding sites (39). However, unlike GR^null^ mice, GR^dim^ animals are viable and normal with respect to the major physiological GR-mediated functions (54), although more susceptible to inflammation (57). In a model of antigen-induced arthritis using GR^dim^ mice, it was shown that GR dimerization is necessary for the anti-inflammatory effects of GC by suppressing Th1 and Th17 cells activity (58). Considering other GR mutations, it was observed that (a) heterozygous GR knockout mice, in which GR expression is reduced by half, were less sensitive to dexamethasone therapy in experimental autoimmune encephalomyelitis (59); (b) mice with selective GR depletion in T cells (GR^lck-Cre^) succumb to toxoplasma infection due to increased TNF and IFN-γ production by Th1 cells (60); and (c) mice with selective GR depletion in the thymocytes showed loss of the adaptive immune response and were immunocompromised (61).

## GR-Mediated Mechanisms of Action

Glucocorticoid effects usually depend on its interaction with GR in the cytoplasm to trigger a variety of cell responses that culminate in several changes in the whole body (62). Although most cells have similar GC response machineries, steroids exhibit varied effects in different tissues (63). This might be attributed to GR ubiquitous expression (except in red blood cells), to the numerous mechanisms that alter its function, and to the enormous assortment of GR-binding sites and their availability. Binding site availability depends on chromatin state, which is specific for each tissue and cell type. Also, GC concentration may determine which GREs are occupied by GR. Therefore, chromatin accessibility and GREs distinct sensitivity also help to explain why GC trigger different effects in different tissues (33). By using different chromatin immunoprecipitation sequencing, it was shown that C/EBPβ maintains chromatin accessibility to facilitate selective GR binding to GREs in liver tissue (64), another evidence of cell type-specific GR-induced transcription. Intracellular GR location, interaction with other proteins, binding capacity and sensitivity to GC and other modulators, receptor degradation rate, and intracellular density directly interfere with GC efficacy. Finally, the effects triggered by the GC-GR complex are tissue and cell type dependent and vary with the posttranslational modifications that will affect the receptor according to the host condition (19, 55).

The classical mechanism of action of nuclear receptors involves gene transcription (transactivation) or direct or indirect impairment of transcription of other TFs (transrepression) (Figure 1: 2). Currently, the most accepted transactivation model involves GR dimerization after binding of GC-GR monomeric complexes to GREs (see Figure 1A). This induces GR conformational changes to recruit cofactors, like histone acetyl transferases and C/EBPβ, that change the chromatin state facilitating gene expression (64–66). The type of dimer that will be formed and which cofactors will be recruited seem to be determined by the gene sequence in question (67, 68). Interestingly, it was recently shown that monomeric GR interacts with a half-site motif and drives transcription (see Figure 1E) in liver and primary macrophages, being more prevalent than homodimer binding under physiological conditions and being essential for those tissue-specific functions. Exogenous GC appear to favor gene expression by the binding of GR homodimers and disruption of GR monomeric binding from their half-site motifs (53). This discovery calls for a revision of transactivation mechanisms and other possible transrepression mechanisms involving monomeric GR binding to half-sites (Figures 1B–E).

Protein–protein interaction of GR with other TFs is an important mechanism of direct transrepression known as tethering mechanism. Monomeric GR can crosstalk with another TF (Figure 1F) or directly interact with critical points of TFs before they bind to its responsive element on DNA (Figure 1J). In this case, best exemplified for the nuclear transcription factor kappa-B (NF-κB), interaction may occur in the cytoplasm, preventing NF-κB activation and its nuclear translocation, or in the nucleus, impairing DNA NF-κB-binding capacity (62, 69). Nonetheless, GR monomers can also repress TFs even if they are already bound to DNA (70) (see Figures 1H,I) and can compete for an overlapping binding site (Figure 1G). Therefore, GR can regulate the expression of different TFs such as NF-κB, AP-1, STATs, Oct, NF-1, C/EBP, COUP-TFII, PPARs, and LXR (71–77).

Glucocorticoid receptor binding to DNA can also suppress gene expression by interacting with novel described negative GREs (nGREs). Recently, an extensive conserved family of negative palindromic GREs (IR nGREs) was found to form a repressing complex through association of SMRT/NCoR corepressors and HDACs, mediating transrepression by the direct binding of agonist-bound GR (78). Evidence showed that two GR monomers can bind to nGREs with inverted polarity if compared on how they bind to GREs (Figure 1L). This kind of monomer binding induce a unique conformational change that, together to the recruitment of corepressors, guarantee the presence of single monomers of GR bound to nGREs (79). As nGREs are near to responsive elements of other TFs, that binding can allosterically prevent the binding of other factors to DNA and recruit corepressors (62, 78) (see Figure 1K).

Indirect modes of reducing gene expression involve GC-induced expression of inhibitory proteins such as glucocorticoid-induced leucine zipper (GILZ) protein *via* modulation of MAPK pathways (16, 80). GILZ protein binds to the p65 subunit of NF-κB in T-cells and macrophages, impairing gene transcription and suppressing inflammation (81, 82). In addition, Lethe, a pseudogene non-coding RNAs induced by cytokines (IL-1β, TNF-α) and GC, might be involved in the negative feedback NF-κB signaling to control inflammation (83).

Since it was believed that GC side effects occur through transactivation mechanism, researchers became interested in identifying SGRMs to enhance GC therapeutic effects with fewer side effects (20, 21, 49, 84). Compound A (CpdA) is a non-steroidal ligand analog that binds GR with high affinity and induces its nuclear translocation. However, it mostly triggers transrepression tethering mechanisms with NF-κB (20, 85, 86) and/or binding to nGREs (79), while inhibiting GR dimerization and preventing dimerization-dependent side effects such as hyperglycemia (79, 85). GR unique conformation upon CpdA binding may impair receptor phosphorylation, which is the suggested mechanism why CpdA does not induce GC resistance and maintain its efficacy even after long periods of administration (86). Unfortunately, CpdA also present a limited therapeutic window due to its toxic effects in higher concentrations (84). *In vitro* studies with CpdX, a novel SEGRM, demonstrated that it is efficient in decreasing inflammation through tethering mechanism, albeit not inducing GRE transactivation and IR nGRE transrepression (87). *In vivo* studies are necessary to reveal the clinical value of CpdX. Compound C108297 presents agonistic and antagonistic properties in the rat brain (88) and can simultaneously prevent diet-induced obesity and inflammation (89). Although promising, it is still unclear whether C108297 is an antagonist or a partial agonist and if it induces a unique conformational of the GR-LBD or a two-state agonist conformation (88). Therefore, more studies are needed to better understand the signaling pathways involved in C108297 effects. Moreover, since new evidence suggests that those agonists or modulators do not necessarily need to be a GR ligand, SEGRMs concept is still in debate (90).

## GR Isoforms and Posttranslational Modifications

There are two major GR isoforms that differ only in their *C*-terminal regions, GRα and GRβ (subtypes of each isoform and other isoforms will not be discussed due to space limitation). The DBD is conserved across the nuclear receptor family and consists of two zinc fingers motifs important to GR dimerization and tandem GRE binding. GRα is the classic receptor responsible for GC actions, whereas GRβ does not bind GC and its biological relevance is still uncertain. It has been suggested that GRβ acts as a negative regulator of GRα isoform (see Figure 1M), because it can bind to GREs but does not induce its transcription (91–94). GRβ has an intrinsic transcriptional activity in non-GC-regulated genes (95). Recently, a pro-inflammatory role for GRβ was shown in the liver tissue of obese mice, suggesting that steatosis correlates to GRβ increased expression in adipose and liver tissues. The same study showed that GRβ antagonizes GC-induced signaling through GRα during fasting (96). So far, this is the first study showing a pro-inflammatory role for GRβ. More studies should focus in unveiling the biological relevance of GRβ in other tissues and cofactors of its singular signaling pathway.

Several posttranslational modifications also play an important role in enhancing or decreasing GR functionality to confer distinct biological functions (see Figure 1: 6). Examples include phosphorylation, acetylation, ubiquitination, methylation, nitrosylation, nitration, and SUMOylation [revised in Ref. (19, 97)]. Due to space limitations, we will focus on recent data about some of them. Nitrosylation of specific cysteine residues decreases GR binding capacity (98, 99) and increases resistance to GC action (100, 101), while tyrosine nitration residues favor nuclear translocation and receptor activity (102). However, exogenous NO seems to activate the endothelial cell GR (103). NO effects very much depend on its concentration and compartmentalization, and this is probably the reason for divergent results. Regarding to SUMOylation, it is related to stabilization, location, and transcriptional activity of GR, typically increasing it (104). However, recent reports demonstrated that SUMOylation is mandatory for GC-dependent transrepression mediated by IR nGREs. SUMOylation of GR lysine residues (mouse: K310 and human: K293) within the NTD is essential for the assembly of the repressive complex SMRT/NCoR-1-HDAC3 (105). The same SUMOylation site is essential for the tethering transrepression mechanism mediated by NF-κB/AP-1, which needs the formation of a GR small ubiquitin-related modifiers (SUMOs)–SMRT/NCoR1-HDAC3 repressing complex (87).

## Ligand-Independent Activation of GR

Beyond the consensus that steroidal or analog binding induces GR nuclear translocation, several evidences have shown that GR can be activated in the absence of ligands (30, 106–110). Sodium arsenite and dinitrophenol (31), some conditions such as elevated pH and temperature, and shear stress (111) can induce GR nuclear translocation in a ligand-independent manner (31, 111, 112) (Figure 1: 1). Interestingly, GR nuclear translocation induced by shear stress does not depend on ligands or intact cytoskeleton (113), but it is related to the nuclear lamina (114).

Unliganded GR acts as a positive regulator of the tumor suppressor gene BRCA1. This beneficial effect is lost upon addition of ligand, suggesting unliganded GR displacement from BRCA1 promoter in response to steroids decreases BRCA1 expression and increases the risk for breast cancer (109). That fact might be related to endogenous monomeric GR binding to half-site motifs, as already discussed. Transient transfection and GR overexpression in GR-deficient cells (COS-1) induced nuclear GR dimerization, GR binding to DNA, and transcription in the absence of exogenous GC (30) (see Figures 1: 1 and 2). Moreover, TNF-α can induce ligand-independent activation of GR pathways in COS-1 and epithelial cells, leading to decreased levels of IL-6 and IL-8 (108). However, the mechanisms of ligand-independent GR activation are not completely understood. One report suggests that GR phosphorylation at Ser-211 is enough for ligand-independent activation (107), whereas another report suggests that phosphorylation at Ser-134 also can induce ligand-independent GR nuclear translocation as part of the cellular stress pathway (115), but other mechanisms might be involved.

## Non-Genomic Mechanisms of Action

Several GC effects occur within a few seconds or minutes after administration, evidencing a non-genomic mechanism of action. This mechanism is particularly important in the vascular system and in dampening inflammation (62, 116) and usually involves activation of non-cytoplasmic GR or even GR-independent pathways (see Figures 1: 3 and 4). Nevertheless, activation of cytoplasmic GR cannot be excluded (33).

Membrane-bound receptors (mGR) have distinct properties if compared to cytoplasmic GR and are more related to intracellular signaling pathways mediated by G-protein-coupled receptors (117, 118). Although some studies suggest that mGR is a GRα isoform (117), others claim that it is in fact a GRγ isoform (119) (Figure 1: 3). Importantly, high doses of GC can act independently of GR by increasing second messengers, such as inositol-3-phosphate, cyclic adenosine monophosphate, and calcium ion (8, 20, 120, 121) (Figure 1: 4). The presence of GR in human mitochondria (122) and similar GRE sequences in mitochondrial DNA (123) were identified long time ago. Moreover, direct GC-stimulated mitochondrial transcription mediated by mitochondrial GR was also demonstrated in hepatocarcinoma cells (124). It was recently suggested that GRγ isoform resides in mitochondria and is related to cell energy metabolism regulation in a ligand-independent manner (119) (see Figure 1: 5).

Protein–protein interaction between ligand-activated GR and the regulatory subunit (p85α) of phosphoinositol-3-kinase in endothelial cells activates the protein kinase Akt, which phosphorylates and activates NO synthase-3. This mechanism would explain the rapid and transient cardiovascular protective effect of high GC doses in myocardial emergencies. A rapid decrease in peripheral resistance and blood pressure is observed minutes after GC administration, accompanied by an increase in coronary and cerebral blood flows (116). Since rapid vasoconstriction and changes in bronchial blood flow are observed few seconds after inhaled GC administration in asthmatic attacks, it suggests that non-genomic effects also may vary according to the tissue (125). Furthermore, endothelial cell stimulation with dexamethasone rapidly activates ERK and kinase c-Jun *N*-terminal MAPK to produce reactive oxygen species and activate TFs (126).

## Summary and Future Directions

In summary, recent data have added new layers of complexity to GR signaling pathways. Clearly GR signaling does not depend only on ligand binding, and its classical mechanisms of action need further revision. Given the physiological and clinical importance of GC and their side effects, it is essential to further investigate alternative GR signaling pathways and their respective biological relevance. This might help to develop new strategies to take benefit of it as well as to improve GC or analogs efficacy with minimal side effects.

## Author Contributions

KS and JA discussed the structure of the manuscript. KS wrote the initial draft and created the figure. KS and JL worked to finish the revised manuscript, and JA did the final review prior to submission. KS, JL, and JA exchanged several revisions until the final manuscript was agreed upon all the authors.

## Conflict of Interest Statement

The authors declare that the research was conducted in the absence of any commercial or financial relationships that could be construed as a potential conflict of interest.
